# Functional Roles of the Dimer-Interface Residues in Human Ornithine Decarboxylase

**DOI:** 10.1371/journal.pone.0104865

**Published:** 2014-08-20

**Authors:** Chien-Yun Lee, Yi-Liang Liu, Chih-Li Lin, Guang-Yaw Liu, Hui-Chih Hung

**Affiliations:** 1 Department of Life Sciences, National Chung-Hsing University, Taichung, Taiwan; 2 Graduate Institute of Biotechnology, National Chung-Hsing University, Taichung, Taiwan; 3 Molecular and Biological Agricultural Sciences Program, Taiwan International Graduate Program, Academia Sinica, Taipei, Taiwan; 4 Institute of Microbiology and Immunology and Division of Allergy, Immunology and Rheumatology, Chung Shan Medical University and Hospital, Taichung, Taiwan; 5 Institute of Medicine, Chung Shan Medical University, Taichung, Taiwan; 6 Institute of Genomics and Bioinformatics, National Chung-Hsing University, Taichung, Taiwan; 7 Agricultural Biotechnology Center (ABC), National Chung-Hsing University (NCHU), Taichung, Taiwan; Griffith University, Australia

## Abstract

Ornithine decarboxylase (ODC) catalyzes the decarboxylation of ornithine to putrescine and is the rate-limiting enzyme in the polyamine biosynthesis pathway. ODC is a dimeric enzyme, and the active sites of this enzyme reside at the dimer interface. Once the enzyme dissociates, the enzyme activity is lost. In this paper, we investigated the roles of amino acid residues at the dimer interface regarding the dimerization, protein stability and/or enzyme activity of ODC. A multiple sequence alignment of ODC and its homologous protein antizyme inhibitor revealed that 5 of 9 residues (residues 165, 277, 331, 332 and 389) are divergent, whereas 4 (134, 169, 294 and 322) are conserved. Analytical ultracentrifugation analysis suggested that some dimer-interface amino acid residues contribute to formation of the dimer of ODC and that this dimerization results from the cooperativity of these interface residues. The quaternary structure of the sextuple mutant Y331S/Y389D/R277S/D332E/V322D/D134A was changed to a monomer rather than a dimer, and the *K*_d_ value of the mutant was 52.8 µM, which is over 500-fold greater than that of the wild-type ODC (ODC_WT). In addition, most interface mutants showed low but detectable or negligible enzyme activity. Therefore, the protein stability of these interface mutants was measured by differential scanning calorimetry. These results indicate that these dimer-interface residues are important for dimer formation and, as a consequence, are critical for enzyme catalysis.

## Introduction

Ornithine decarboxylase (ODC, EC 4.1.1.17) is universally found in organisms ranging from bacteria to humans. ODC catalyzes the pyridoxal 5-phosphate (PLP)-dependent decarboxylation of ornithine to putrescine, and it is the first and the rate-limiting, enzyme in polyamine biosynthesis [1]–[4]. ODC and cellular polyamines play significant roles in numerous biological functions, including embryonic development, the cell cycle, and cell proliferation, differentiation and apoptosis [5]–[11]. Because of their biological roles, polyamines have been linked with several cancers [5], [12]–[21]. Furthermore, because ODC activity and the cellular levels of polyamine are crucial for cell proliferation [9] and are critical for the initiation and progression of neoplastic diseases [2], [22], ODC has been recognized as an oncogenic enzyme. Thus, ODC inhibitors and negative regulators of the polyamine pathway could be beneficial for the treatment of many cancers [3], [7].

ODC is a homodimer with 2-fold symmetry [23], [24], and each subunit has its own active site. Dimerization is critical for the enzymatic function of ODC because the active site of each subunit is located at the dimer interface [25], [26]. Structural data demonstrate that the two active sites of ODC are formed by the N-terminus of one subunit, which contains the residues involved in PLP interactions, and the C-terminus of the other subunit, which contains the residues involved in substrate binding [23], [27]–[34].

The cellular levels of ODC are highly regulated through an exclusive ubiquitin-independent pathway [5]. The enzyme undergoes degradation by directly interacting with its regulatory protein antizyme (AZ) [35]. The binding of AZ to ODC promotes the dissociation of the ODC homodimers and the subsequent formation of an AZ-ODC heterodimer, which is enzymatically inactive [36]–[39]. Thus, AZ inactivates ODC by forming inactive AZ-ODC heterodimers. In addition, AZ targets ODC for degradation via the 26S proteasome [40]–[42]. Therefore, the primary role of AZ is to regulate polyamine metabolism through inhibition of ODC activity and polyamine transport, thus restricting polyamine levels [5], [35], [43], [44]. Because increased ODC activity is associated with most human malignancies [2], AZ has been suggested as a tumor suppressor; thus, AZ has potential in the development of protein drugs. Our recent studies have indicated that a minimally functional AZ peptide, AZ_95-176, inhibits ODC enzyme activity as effectively as the full-length AZ protein [45].

A regulatory protein termed the antizyme inhibitor (AZI) is an antagonist of AZ. AZI positively regulates ODC, which is down-regulated by AZ. AZI is homologous to ODC but does not show decarboxylase activity [46]. AZI binds to AZ more tightly than ODC and rescues ODC from the AZ-ODC complex [47]. The dissociation constant (*K*_d_) of the AZ-ODC complex is approximately 0.2 µM, while that of the AZ-AZI complex is approximately 0.02 µM. Thus, there is a 10-fold difference between the binding affinities of AZ-AZI and AZ-ODC [48]. As a result, AZI restores ODC activity [47]–[49] and prevents proteasomal degradation of ODC. The factors governing the differential binding affinities of human ODC and AZI have been identified [48]; differences in residues 125 and 140 of ODC and AZI are responsible for the differential AZ-binding affinities [48].

Structural studies indicate that both ODC and AZI form dimers when crystallized [23], [50]; however, ODC exists as a dimer in solution, with a *K*_d_ value of approximately 0.18 µM, whereas AZI exists as a monomer-dimer equilibrium, with a *K*_d_ value of approximately 84 µM. Thus, the self-association of the subunits of these two proteins differs by greater than 400-fold [49]. The AZI dimer has fewer interactions than the ODC dimer and lacks symmetric interactions between residues at the dimer interface [50]. We have identified the critical amino acid residues responsible for the difference in dimer formation between ODC and AZI.

Here, we continue to discuss how ODC forms such a stable dimer and the factors determining dimer formation. In addition, we discuss the role of these interface residues in protein stability. Previous studies of trypanosomal ODC have revealed that no single, unique dimer-interface residue is critical for the dimerization [26]. In this study, a series of dimer-interface mutants of ODC were created. Kinetic experiments and biophysical studies, including analytical ultracentrifugation (AUC) and differential scanning calorimetry (DSC), were employed. Our data indicated that ODC dimerization results from the cooperativity of these interface residues. In addition, we propose that these dimer-interface residues are also critical for protein stability and enzyme activity.

## Results

**Figure 1:**
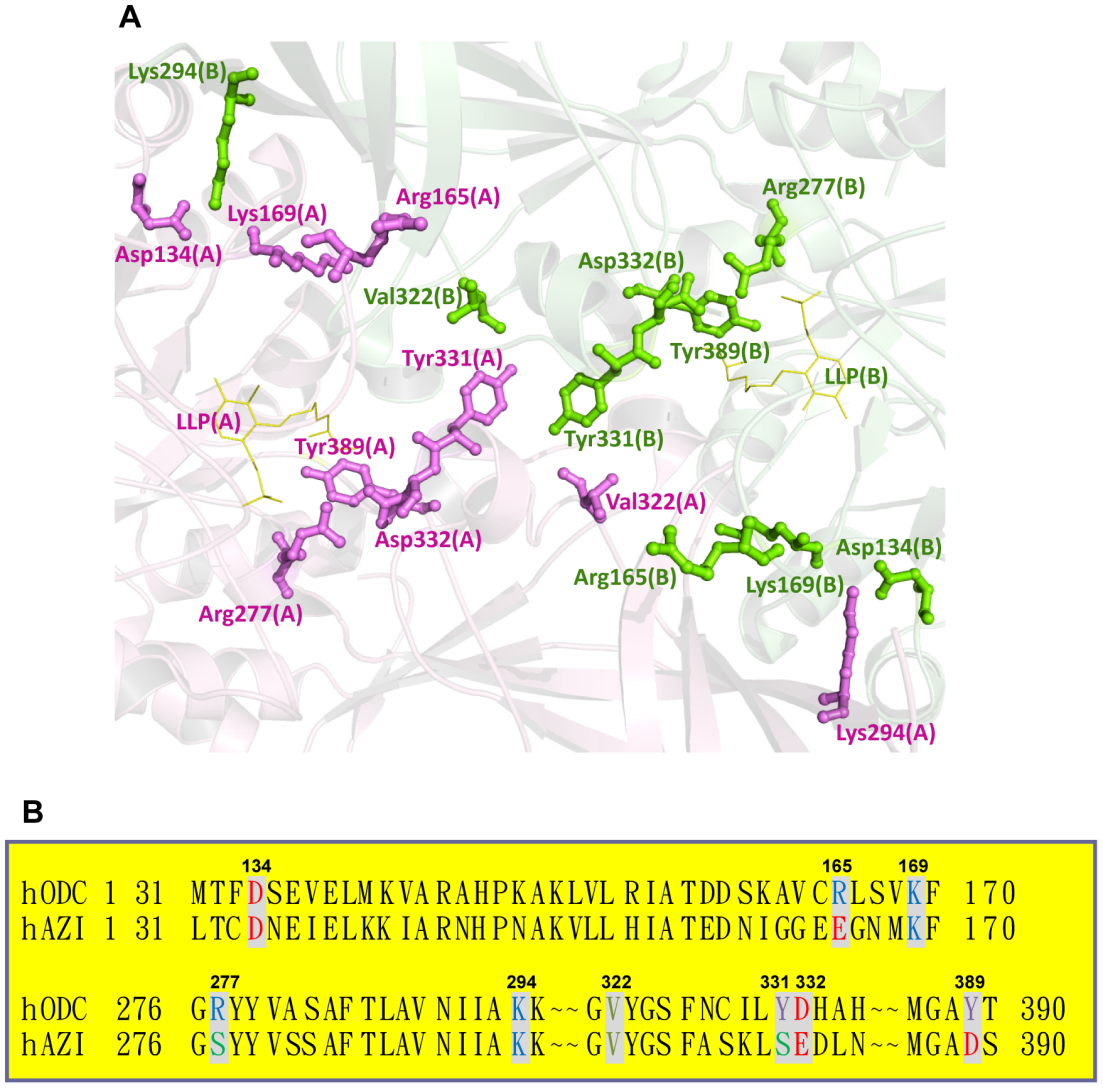
Amino acid residues at the dimer interface of human ornithine decarboxylase. (A) The dimer-interface residues and the cofactor PLP analog, LLP (PDB code 1D7K), at the dimer interface of ODC. These residues are shown as a ball-and-stick model; the residues of one subunit are shown in blue, and those of the other subunit are shown in pink. This figure was generated using PyMOL [57]. (B) Pairwise sequence alignments between human ODC and AZI at the dimer interface.

The structure of human ODC indicates that some amino acid residues in the dimer interface may play significant roles in either dimer formation or enzyme activity (Figure 1A). Previous studies have indicated that ODC exists as a stable dimer, with a dissociation constant (*K*_d_) of 0.18 µM, whereas AZI is in a rapid equilibrium between the monomer and the dimer, with a *K*_d_ value of 84 µM [49]. In this study, we explored the functional roles of the dimer-interface residues that may contribute to dimerization of the enzyme, enzyme activity and protein stability. The five distinctive amino acid residues in ODC were changed to the corresponding residues in AZI: R165E, R277S, Y331S, D332E and Y389D (Figure 1B). Four conserved residues were mutated as follows: Val322 was changed to aspartate (V322D), and Asp134, Lys169 and Lys294 were each changed to alanine (D134A, K169A and K294A).

### The kinetic properties of wild-type and dimer-interface mutants of human ODC

First, the kinetic parameters of wild-type (WT) human ODC and the 9 enzymes with the single mutations were determined, and the *k*_cat_ values of the WT and dimer-interface mutants found to be quite different (Table 1). The *k*_cat_ of the WT was approximately 225 min^−1^. Compared with the WT, the non-conserved mutants (R277S, Y331S and Y389D) and the conserved mutants (D134A, K169A and K294A) showed low *k*_cat_ values and decreased catalytic activity. The *k*_cat_ values of K169A, R277S, K294A and Y331S were 27, 21, 33 and 40 min^−1^, respectively, which were only 10% to 27% of the WT turnover number. Furthermore, the enzyme activities of D134A, V322D and Y389D were negligible, as indicated by their *k*_cat_ values. These results demonstrate the significance of these dimer-interface residues for enzyme activity. Other single mutants, such as R165E and D332E, displayed *k*_cat_ values (257 and 177 min^−1^, respectively) comparable to that of WT, suggesting that these residues were much less important for the function of the enzyme.

**Table 1:** Kinetic parameters of human ornithine decarboxylases.

ODC Variants	*K*_m,ornithine_ (mM)	*k*_cat_ (min^−1^)
WT	0.37±0.09	225±16
D134A	3.54±1.05	4.2±0.3
R165E	0.55±0.09	257±14
K169A	0.35±0.06	27±1.5
R277S	0.37±0.16	21±1.2
K294A	0.28±0.02	33±0.6
V322D	0.43±0.05	4.5±0.5
Y331S	0.39±0.09	40±3.0
D332E	0.42±0.10	177±13
Y389D	N.D.	N.D.
Y331S/Y389D	N.D.	N.D.
Y331S/Y389D/R277S	N.D.	N.D.

*N.D. indicates that the enzyme activity was not detectable.

### Size distribution analysis of the WT and dimer-interface mutants of human ODC

**Figure 2:**
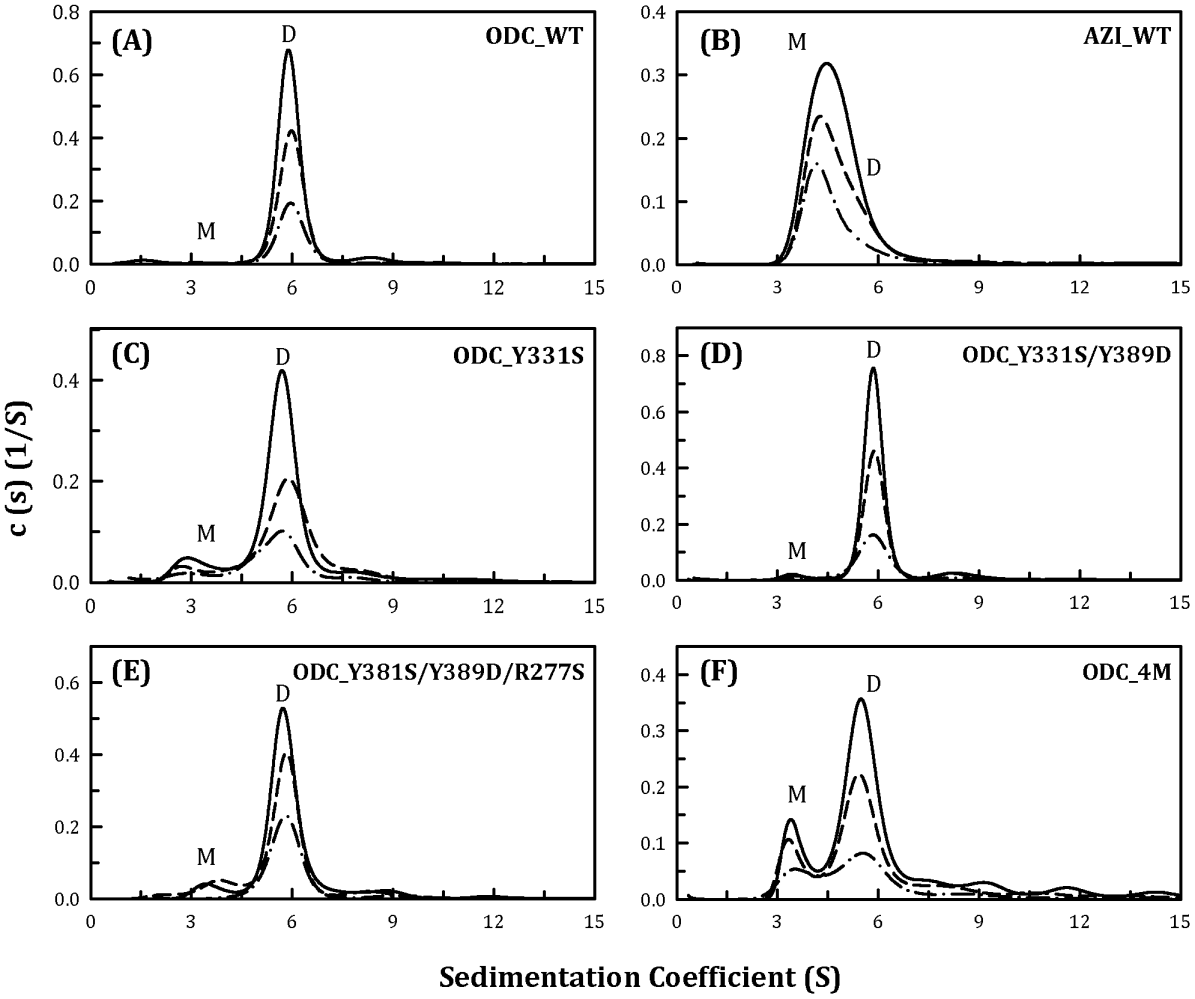
Continuous sedimentation coefficient distribution of the human ODC_WT and the single, double, triple and quadruple mutants. The three protein concentrations (from top to bottom) were 0.3, 0.6 and 0.9 mg/ml. (A) ODC_WT; (B) AZI; (C) ODC_Y331S; (D) ODC_Y331S/Y389D; (E) ODC_Y331S/Y389D/R277S; and (F) ODC_Y331S/Y389D/R277S/D332E (ODC_4M). M, monomer; D, dimer.

The size distributions of the WT and the 9 single mutants of human ODC were examined (Figure 2A for WT, Figure 2C for Y331S and Figure S1 for the other mutants), and the dissociation constants of these single mutants were determined by global fitting of the sedimentation velocity data (Table 2). The *K*_d_ value of the WT ODC was 0.1 µM. The sedimentation plots and the *K*_d_ values of the ODC single mutants indicated that no individual residue significantly interrupted the ODC dimer interface. Most single mutants still successfully maintained dimers with a *K*_d_ value similar to that of the WT (Figure S1; Table 2). The single mutant Y331S displayed a 10-fold higher *K*_d_ than the WT; and a small amount of monomers was present. The single mutants V322D and Y389D had *K*_d_ values approximately 6-fold greater than that of the WT (Table 2). These data imply that interacting forces exist among these particular residues and that these forces may drive dimer formation in ODC. Thus, double, triple, quadruple, quintuple and sextuple mutants were subsequently created in various combinations.

**Table 2:** Dissociation constants for the dimer-monomer equilibrium of human ornithine decarboxylases.

ODC Variants	*K*_d_ (µM)
WT	0.10±0.001
D134A	0.12±0.012
R165E	0.11±0.001
K169A	0.30±0.008
R277S	0.08±0.006
K294A	0.10±0.008
V322D	0.56±0.012
Y331S	1.10±0.008
D332E	0.03±0.007
Y389D	0.69±0.005
Y331S/Y389D	0.16±0.001
Y331S/Y389D/R277S	1.03±0.004
Y331S/Y389D/R277S/D332E	15.9±0.08
Y331S/Y389D/R277S/D332E/R165E	13.5±0.45
Y331S/Y389D/R277S/D332E/D134A	13.3±0.04
Y331S/Y389D/R277S/D332E/K169A	1.49±0.04
Y331S/Y389D/R277S/D332E/K294A	0.26±0.06
Y331S/Y389D/R277S/D332E/V322D	25.2±0.03
Y331S/Y389D/R277S/D332E/V322D/D134A	52.8±0.50
AZI	72.3±0.70

**Figure 3:**
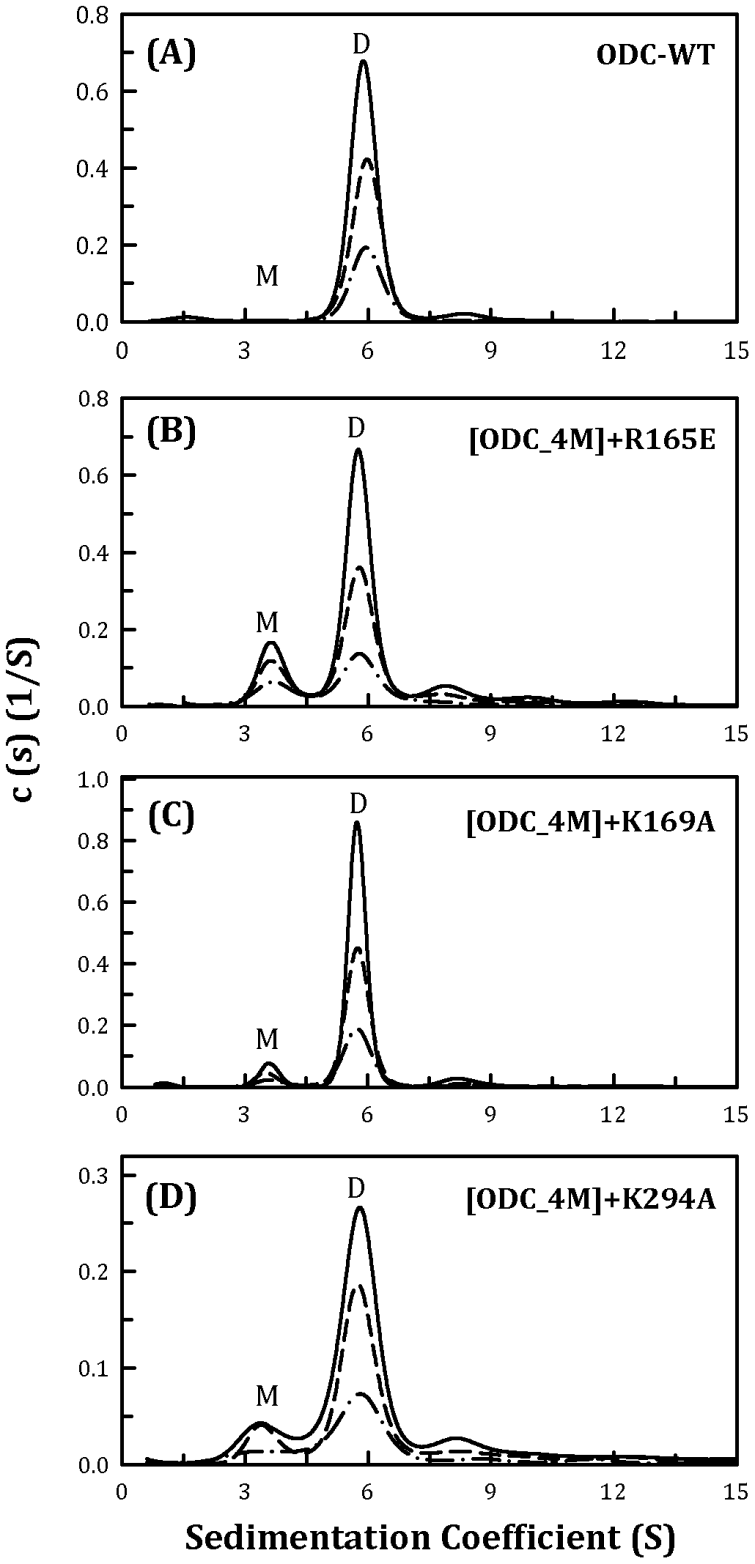
Continuous sedimentation coefficient distribution of the human ODC_WT and the ODC_4M mutants with R165E, K169A and K294A. The three protein concentrations (from top to bottom) were 0.3, 0.6 and 0.9 mg/ml. (A) ODC_WT; (B) [ODC_4M]+R165E; (C) [ODC_4M]+K169A; and (D) [ODC_4M]+K294A. M, monomer; D, dimer.

**Figure 4:**
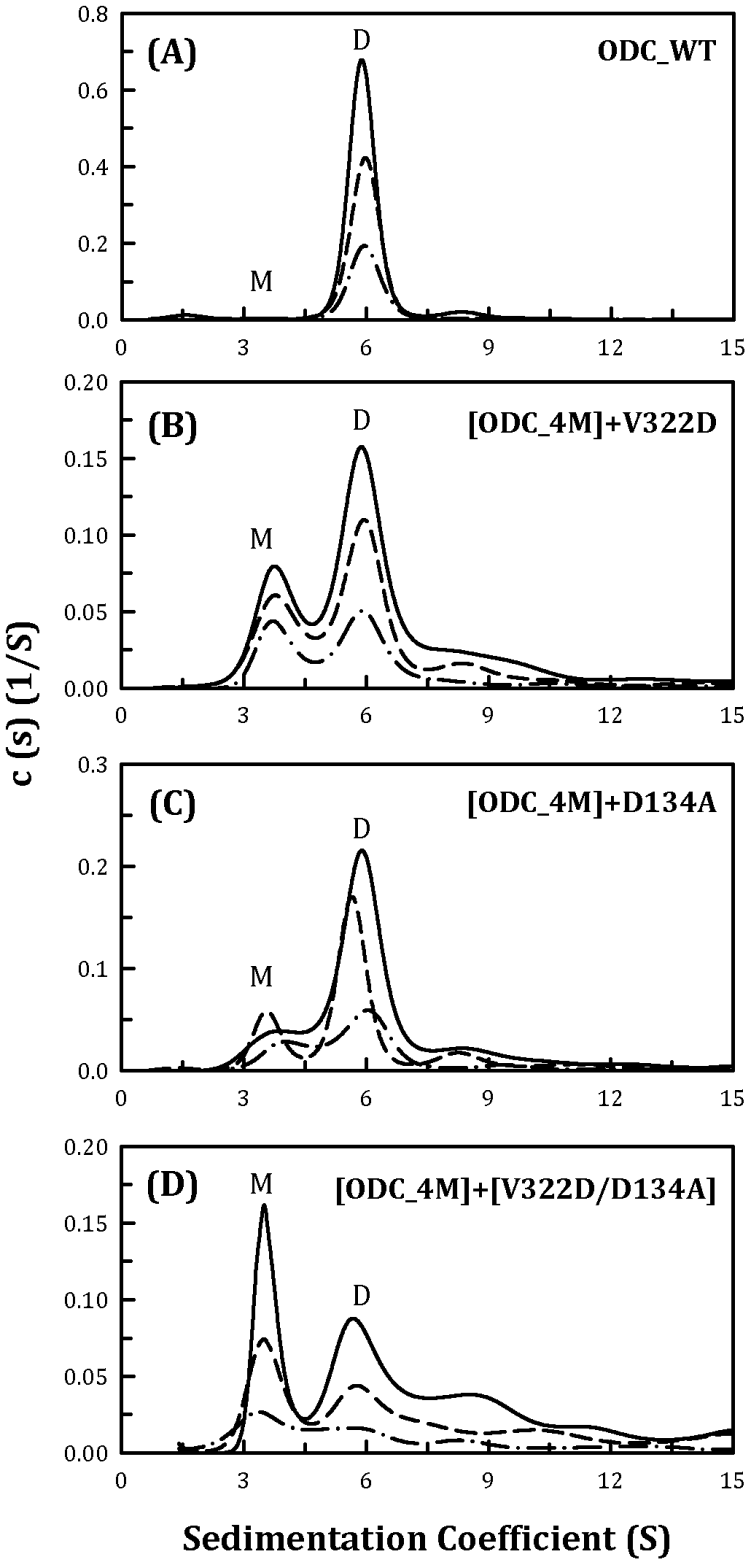
Continuous sedimentation coefficient distribution of the human ODC_WT and the ODC_4M mutants with D134A and V322D. The three protein concentrations (from top to bottom) were 0.3, 0.6 and 0.9 mg/ml. (A) ODC_WT; (B) [ODC_4M]+V322D;(C) [ODC_4M]+D134A; and (D) [ODC_4M]+[V322D/D134A]. M, monomer; D, dimer.

*The *K*_d_ value was derived from global data fitting of the sedimentation velocity using three different ODC concentrations (Figures 2–4 and Figure S1).

We have shown that an AZI quadruple mutant, AZI_S331Y/D389Y/S277R/E332D, is a stable dimer, similar to the dimeric ODC, with a dissociation constant of approximately 0.1 µM. Figure 2 shows the size-distribution plots for ODC_WT, ODC_Y331S, ODC_Y331S/Y389D, ODC_Y331S/Y389D/R277S and ODC_Y331S/Y389D/R277S/D332E (ODC_4M). Although the single mutation of Tyr331 to Ser (ODC_Y331S) or Tyr389 to Asp (ODC_Y389D) in ODC resulted in a *K*_d_ value (1.1 and 0.69 µM, respectively, Table 2) larger than that of the WT (0.1 µM), there was no difference in the *K*_d_ values between the WT and ODC_Y331S/Y389D (0.1 and 0.16 µM, respectively, Table 2). ODC_Y331S/Y389D/R277S displayed a small shift in the monomer-dimer equilibrium (Figure 2E), with a *K*_d_ value of 1.03 µM (Table 2), which was very similar to that of ODC_Y331S (Figure 2C). The ODC dimer clearly dissociated when D332E was added to the ODC_Y331S/Y389D/R277S triple mutant. However, although the *K*_d_ value of the ODC_S331Y/D389Y/S277R/D332E quadruple mutant was 160-fold greater than that of the WT (15.9 µM versus 0.1 µM, Table 2), the quadruple mutant still existed predominantly in dimeric form (Figure 2F). Thus, although the AZI quadruple mutant S331Y/D389Y/S277R/E332D behaved as a dimer, the corresponding ODC quadruple mutant did not primarily exist in its monomeric form. We therefore continued to examine other interface residues that could affect ODC dimerization appreciably.

Three cationic amino acid residues in ODC, Arg165, Lys169 and Lys294, were substituted individually with Glu, Ala and Ala in ODC_4M. The quintuple mutant [ODC_4M]+R165E showed a pattern similar to that of ODC_4M (Figure 3B), with a *K*_d_ value of 13.5 µM, which was comparable to that of ODC_4M (Table 2). The other two quintuple mutants, [ODC_4M]+K169A and [ODC_4M]+K294A, did not show a notable shift in the monomer-dimer equilibria (Figure 3, C and D, respectively); their *K*_d_ values were 1.49 and 0.26 µM, respectively (Table 2), which were similar to that of ODC_Y331S (Figure 2C). This result indicated that Arg165, Lys169 and Lys294 have little effect on ODC dimerization.

A conserved hydrophobic amino acid residue, Val322, had an appreciable effect on ODC dimerization. In the current study, Val322 was changed to Asp to interrupt the hydrophobicity at the dimer interface. When V322D was added to ODC_4M, the resulting quintuple mutant clearly showed a monomer-dimer equilibrium (Figure 4B) compared with ODC_4M (Figure 2F); the *K*_d_ of [ODC_4M]+V322D was 25.2 µM (Table 2), which was 1.6-fold larger than that of ODC_4M. This result indicated that Val322 had a positive effect on ODC dimerization.

A conserved amino acid residue at the dimer interface, Asp134, was also examined for its contribution to the dimerization of ODC. The *K*_d_ value of [ODC_4M]+D134A was approximately 13.3 µM (Figure 4C), which was not significantly different from that of ODC_4M (Table 2). However, when both V322D and D134A were added to ODC_4M, the resulting sextuple mutant demonstrated a significant shift from dimer to monomer (Figure 4D); the *K*_d_ value of [ODC_4M]+[V322D/D134A] was 52.8 µM (Table 2), which was over 500-fold greater than that of ODC_WT. For this mutant, the predominant form was the monomer rather than the dimer, further demonstrating the synergistic effect of these interface residues on the formation of the dimer of ODC.

### DSC scanning study of the WT and dimer-interface mutants of human ODC

**Figure 5:**
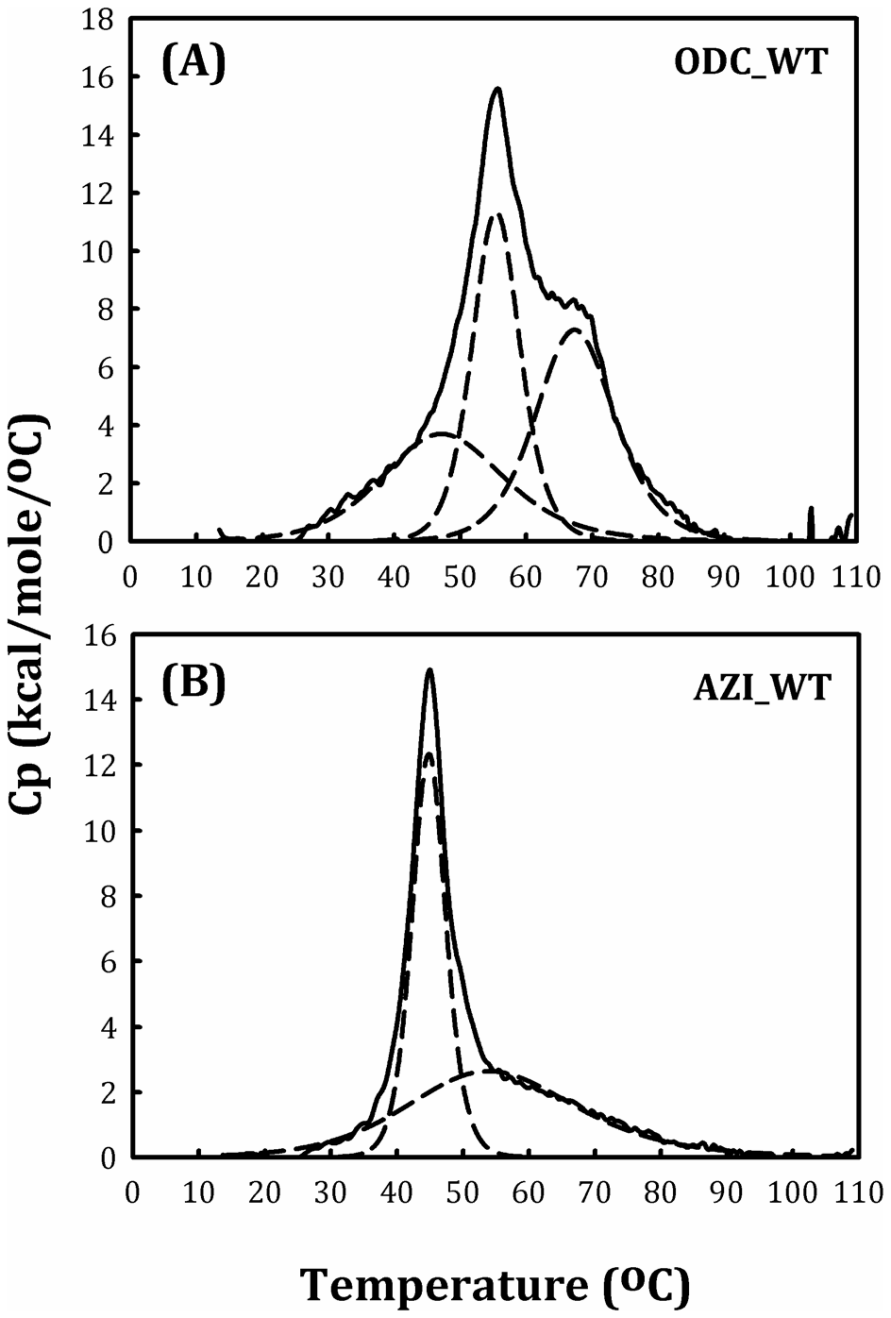
DSC scanning plot of the human ODC and AZI. The protein concentration was 0.75 mg/ml in 30 mM sodium acetate buffer (pH 7.0). (A) ODC_WT and (B) AZI.

We analyzed the protein stability of WT ODC and AZI using the differential scanning calorimetric technique, and the melting temperature values (*T*_m_) are shown in Table 3. The DSC scanning plot of ODC_WT was a multiple curve with several *T*_m_ values (Figure 5A, solid curve); after appropriate fitting, the curve can be divided into three sub-curves with *T*_m_ values of 48, 56 and 67°C (Figure 5A, dashed curves; Table 3). The DSC scanning plot of AZI was also not a simple curve (Figure 5B, solid curve). After curve fitting, we observed a multiple curve consisting of two sub-curves with *T*_m_ values of 45 and 55°C (Figure 5B, dashed curves; Table 3). ODC and AZI have very similar structural folds [50]; however, ODC is a dimer, whereas AZI predominantly exists as monomers (Figure 2A and 2B, respectively). Therefore, one of the three *T*_m_ values of ODC may represent stable subunit-subunit interactions, while the other two may contribute to the conformational stability of the ODC and AZI monomers.

**Table 3:** Melting temperatures of WT and mutant human ornithine decarboxylase.

ODC Variants	*T*_m1_ (°C)	Δ*T*_m1(WT-mut)_	*T*_m2_ (°C)	Δ*T*_m2(WT-mut)_	*T*_m3_ (°C)	Δ*T*_m3(WT-mut)_
WT	48±3.3	0	56±0.1	0	67±0.3	0
R165E	44±1.7	−4	51±0.2	−5	61±0.2	−6
D134A	37±0.6	−11	52±0.3	−4	62±0.4	−5
K169A	41±0.6	−7	55±0.3	−1	62±0.7	−5
R277S	36±1.4	−12	51±0.9	−5	63±0.4	−4
Y331S	40±0.9	−8	61±0.3	−5	68±0.2	+1
D332E	35±5.8	−13	51±3.1	−5	62±1.1	−5
K294A	35±0.6	−13	47±0.7	−9	57±0.5	−10
V322D	34±1.9	−14	47±0.6	−9	58±0.2	−9
Y389D	32±3.8	−16	48±2.4	−8	56±0.4	−11
Y331S/Y389D	35±0.7	−13	46±0.1	−10	55±0.1	−12
Y331S/Y389D/R277S	38±1.6	−10	45±0.2	−11	54±0.1	−13
Y331S/Y389D/R277S/D332E	37±0.3	−11	48±0.2	−8	56±0.1	−11
Y331S/Y389D/R277S/D332E/V322D	-	-	40±0.2	−17	50±0.2	−17
Y331S/Y389D/R277S/D332E/V322D/D134A	-	-	44±0.6	−12	59±0.9	−8
AZI	-	-	45±0.02	-	55±0.3	-

**Figure 6:**
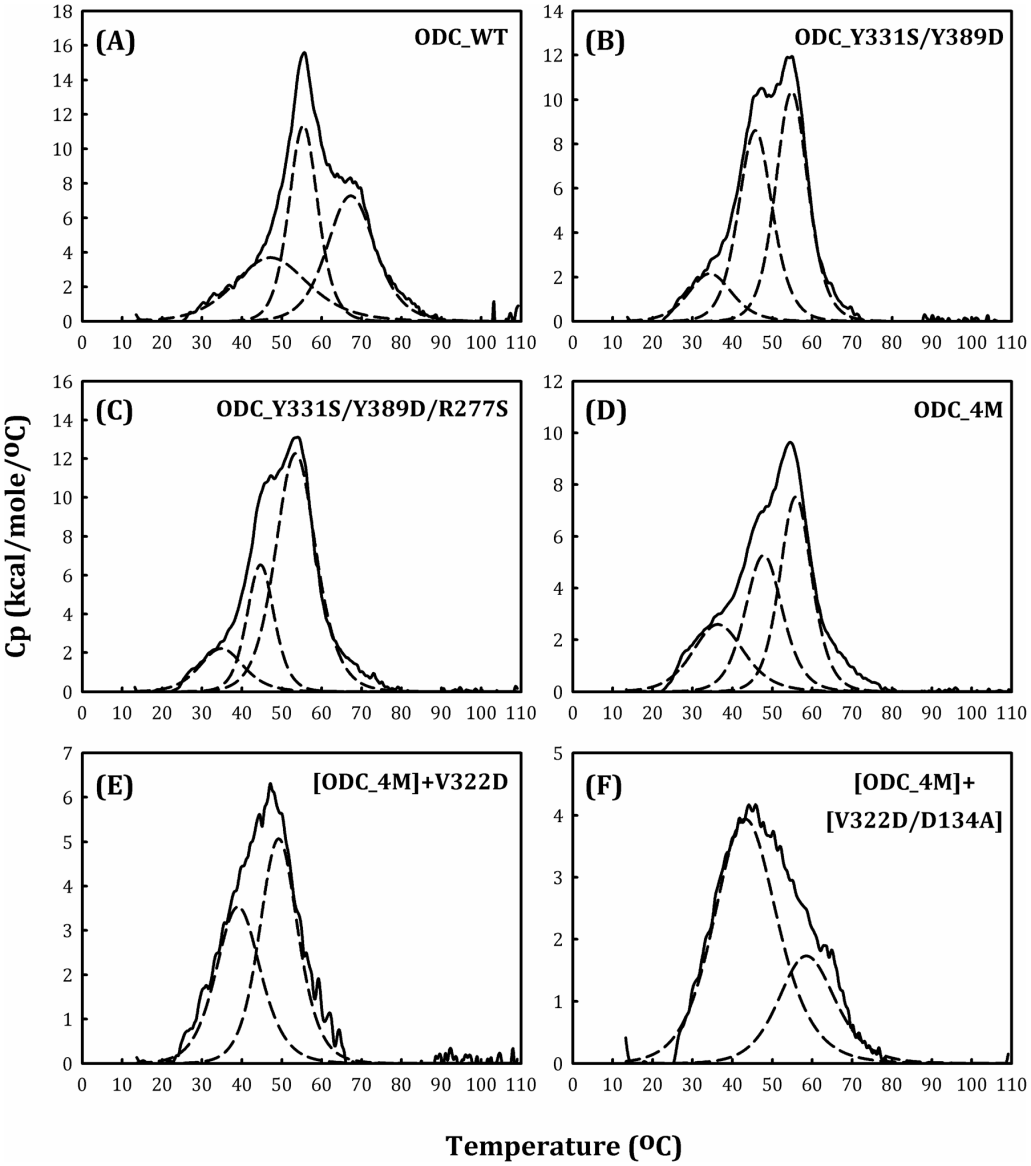
DSC scanning plot of the human ODC_WT and multiple mutants. The protein concentration was 0.75 mg/ml in 30 mM sodium acetate buffer (pH 7.0). (A) ODC_WT; (B) ODC_Y331S/Y389D; (C) ODC_Y331S/Y389D/R277S; (D) ODC_Y331S/Y389D/R277S/D332E (ODC_4M); (E) [ODC_4M]+V322D; and (F) [ODC_4M]+[V322D/D134A].

The *T*_m_ value was derived from the DSC scanning data shown in Figure 5, Figure 6 and Figure S2.

Δ*T*_m(WT-mut)_ refers to the difference in *T*_m_ values between the WT and mutant.

The protein stabilities of the ODC interface mutants were also determined. The DSC scanning plot of the R165E mutant was quite similar to that of the WT (Figure S2B, solid curve), and the *T*_m_ values were approximately 44, 51 and 61°C (Table 3), which were 5°C less than those of the WT. Because the *K*_d_ and kinetic properties (*K*_m_ and *k*_cat_) of R165E were not different from those of the WT, the amino acid substitution of R with E did not have a significant effect on the structure and enzymatic function of ODC.

The single mutants D134A, K169A, R277S, Y331S and D332E each showed three *T*_m_ values; however, one *T*_m_ value (designated as *T*_m1_) was significantly lower than that of the WT by approximately 7–13°C (Table 3). In addition, the patterns of their DSC scanning plots were changed from that of the WT (Figure S2, C–G, solid curve). Although their *K*_d_ values were not significantly altered, their *k*_cat_ values were substantially smaller. Therefore, the *T*_m1_ value of ODC may signify inter-subunit stability, and the decrease in the *k*_cat_ and catalytic activity of the enzyme may be associated with a reduction in *T*_m1_.

The T_m_ values of the single mutants K294A, V322D and Y389D were significantly (8–16 K) lower than that of the WT enzyme (Table 3). Additionally, the patterns of the scanning plots were altered (Figure S2, H–J). In addition, the multiple mutants ODC_Y331S/Y389D and ODC_Y331S/Y389D/R277S showed similar changes in *T*_m_ values (Table 3) and scanning patterns (Figure 6B and 6C, respectively). These results indicated that the overall conformational stability of ODC was noticeably altered by these mutations and that the negligible catalytic activity of these mutant enzymes could be attributed to changes in the enzyme conformation.

The most interesting phenomenon observed in the DSC study was the dramatic difference in the *T*_m_ value between ODC_Y331S/Y389D/R277S/D332E (ODC_4M) and [ODC_4M]+V322D. ODC_4M had three *T*_m_ values, while [ODC_4M]+V322D had only two *T*_m_ values (Figure 6D and 6E, respectively). The *T*_m_ values for ODC_4M were 37, 48 and 56°C, and those for [ODC_4M]+V322D were 40 and 50°C (Table 3). Because the monomer-dimer equilibrium shifted between ODC_4M and [ODC_4M]+V322D, with an increase in *K*_d_ value (15.9 µM and 25.2 µM, respectively, Table 2), the loss of *T*_m1_ indicated dissociation of the ODC dimer into monomers, similar to ODC and AZI (Figure 5). Indeed, the quintuple mutant Y331S/Y389D/R277S/D332E/V322D showed a significant shift in the monomer-dimer equilibrium (Figure 4B) and the absence of *T*_m1_ (Table 3).

An ODC sextuple mutant, [ODC_4M]+[V322D/D134A], had *T*_m_ values of 44 and 59°C (Table 3). The size distribution analysis of this mutant enzyme showed that monomers were predominant at equilibrium, with a *K*_d_ value of 52.8 µM (Table 2). This finding coincided with two *T*_m_ values associated with dissociation of the mutant enzyme.

## Discussion

ODC functions as a dimer, and this form is required for its decarboxylase activity [23], [24]. In this work, we determined the roles of the amino acid residues at the dimer interface. Our results indicated that most of the dimer-interface amino acid residues are important for the stability of the enzyme and contribute to the subunit-subunit interactions and/or the conformational stability of the monomers. The significant effects of certain interface residues on enzyme activity may be attributed to their role in dimer formation. In addition, the data from the AUC and DSC studies illustrate the differences in the dimeric enzyme and its homolog with respect to dimerization, enzyme activity and protein stability.

### Factors that affect the dimerization of ODC

Size-distribution analysis of the ODC interface mutants clearly indicates that dimer formation is a result of the cooperative behavior of the interface residues. The structure of human ODC suggests the importance of the hydrophobic interactions involving Tyr331 and Val322 at the dimer interface. Mutating Y331S or V322D in ODC results in a minimal shift in the equilibrium from the dimeric to the monomeric form, with a *K*_d_ value that is over 5-fold greater than that of the WT. Tyr389 may also be crucial for dimerization because its *K*_d_ value was 7-fold higher than that of the WT (Table 2).

The single mutations of Arg277 and Asp332 showed *K*_d_ values that were smaller than that of the WT (Table 2). However, these mutations contributed to dimerization because the *K*_d_ value of ODC_Y331S/Y389D/R277S/D332E was 100-fold larger than that of ODC_Y331S/Y389D (Table 2). The structure of ODC shows ionic interactions between Arg277 and Asp332 in the same subunit; this ion pair should contribute to formation at the dimer interface.

Three conserved amino acid residues at the dimer interface of ODC and AZI were examined for their contribution to dimer formation. The structure of ODC shows that Asp134, Lys169 and Lys294 compose a region with an electrostatic network. Our data suggest that ionic interactions involving Asp134 influence dimer formation because the *K*_d_ value of ODC_Y331S/Y389D/R277S/D332E/V322D/D134A was 2-fold greater than that of ODC_Y331S/Y389D/R277S/D332E/V322D (Table 2).

### Dimer-interface residues are important for ODC enzyme activity and protein stability

Some of the dimer-interface residues are critical for enzyme catalysis, and mutation of these residues results in loss of enzyme catalysis. Previous studies have shown the importance of Arg277 and Tyr389 in the binding of the cofactor PLP [28], [34]. Thus, it is not surprising that R277S and Y389D show limited enzyme activity (Table 1). In addition, the *K*_d_ value of Y389D is 6.9-fold larger than that of the WT, suggesting its role in the dimerization of ODC. In the DSC study, all three *T*_m_ values of the Y389D enzyme were notably lower than those of the WT. Therefore, Tyr389 may have play a role not only in the binding of PLP but also in dimer formation and structural stability.

Tyr331 and Val322 reside at the dimer interface, and mutations of these two residues, especially the V322D mutation, make the enzyme less active. It is believed that Tyr331 and Val322 do not directly participate in enzyme catalysis. The loss of enzyme activity may be attributed to a small change at the dimer interface because the *K*_d_ values of Y331S and V322D are 11- and 5.6-fold larger than that of the WT. Similarly, Asp134, Lys169 and Lys294 are not catalytic residues and are located far from the active site of ODC. However, mutating these residues is notably detrimental to enzyme activity. In particular, D134A showed limited catalytic activity. Indeed, D134A, K169A, K294A, V322D and Y389D show negligible enzyme activity and markedly low *T*_m_ values, although their dimeric structures are mostly retained. These results indicate that these interface residues are required for the proper conformation of the dimer interface and are critical for enzymatic activity.

In summary, according to the above results, we suggest that drugs that are capable of binding to the dimer interface or interfering with dimerization of the enzyme may have potential as novel ODC inhibitors. Our studies provide information for the rational drug design of ODC inhibitors that bind to the dimer interface with high affinity.

## Materials and Methods

### Expression and purification of recombinant wild-type and mutant human ODC

Human ODC cDNA was cloned into the pQE30 vector (Qiagen, Hilden, Germany), which carries an N-terminal His·Tag sequence useful for purifying overexpressed proteins, and the expression vectors were transfected into the JM109 strain of *Escherichia coli*. Protein expression was induced with 1.0 mM isopropyl-1-thio-β-D-galactoside (IPTG), and the cells were grown at 25°C overnight. Before purification, the cell pellets were dissolved in 25 ml of binding buffer (5 mM imidazole, 500 mM sodium chloride, 30 mM Tris-HCl, pH 7.6) with 2 mM β-mercaptoethanol, 2 mM phenylmethylsulfonyl fluoride (PMSF) and 0.2% Triton X-100. HIS-Select Nickel Affinity Gel (Sigma, St Louis, MO) was used to purify the ODC protein. For the column, 2 ml of the affinity gel was first equilibrated with the binding buffer and then mixed with the cell lysate at 4°C for 30 min. Then, the lysate-Ni-gel mixture was washed with 100 ml of binding buffer and wash buffer (10 mM imidazole, 500 mM sodium chloride and 30 mM Tris-HCl, pH 7.6) to remove unwanted proteins. Finally, the target proteins were removed with elution buffer (250 mM imidazole, 500 mM sodium chloride and 2 mM β-mercaptoethanol in 30 mM Tris-HCl, pH 7.6). After a stepwise wash process, the proteins were eluted and concentrated for subsequent experiments. The purity of all recombinant proteins was determined with SDS-PAGE.

### Site-directed mutagenesis

Site-directed mutagenesis was performed using a QuikChange Kit (Stratagene, La Jolla, CA). This mutagenesis method uses *Pfu* DNA polymerase, which replicates both plasmid strands with high fidelity in a 16–20 cycle PCR reaction. Primers 35–45 bases in length that included the desired mutations were used to specifically amplify the template DNA. The sequences of the mutagenic primers used in this study were as follows:

R165E: 5′-GATGATTCCAAAGCAGTCTGTGAACTCAGTGTGAAATTCGGTGCC-3′; D134A: 5′-GAGTCCAGTAGATGACTTTTGCGAGTGAAGTTGAGTTGATG-3′; K169A: 5′-CTGTCGTCTCAGTGTGGCGTTCGGTGCCACGCTC-3′; R277S: 5′-GCTGAGCCCGGCAGCTACTATGTTGCATC-3′; Y331S: 5′-CATTTAATTGCATACTCTCTGACCACGCACATGTAAAGC-3′; D332E: 5′-TAATTGCATACTCTATGAGCACGCACATGTAAAGCCC-3′; K294A: 5′-GTTAATATCATTGCCAAGGCGATTGTATTAAAGGAACAGACGGGC-3′; V322D: 5′- GTATTATGTGAATGATGGCGACTATGGATCATTTAATTGC-3′; Y389D: 5′-GAAAACATGGGCGCTGACACTGTTGCTGCT-3′; and Y331S/D332E: 5′- CATTTAATTGCATACTCTCTGAGCACGCACATGTAAAGCCCCTTC-3′.

The PCR cycling conditions were as follows: 20 cycles of 95°C for 30 sec, 55°C for 1 min and 68°C for a period equivalent to 2 min/kb of plasmid length. The PCR products were then treated with DpnI to digest the WT human ODC templates. Finally, the nicked DNA with the desired mutations was transfected into the XL-1 *E. coli* strain (Stratagene, La Jolla, CA), and the DNA sequences were checked by autosequencing.

### Enzyme assay and kinetic analysis

The decarboxylase activity of ODC was measured with a CO_2_-L3K assay kit (DCL, Charlottetown, Canada) at 310 K. For continuous measurement of the ODC enzyme activity, the decarboxylation of ornithine was coupled to the carboxylation of phosphoenolpyruvate (PEP) to form oxaloacetate (OAA), which becomes malate following NADH oxidation, according to a previously published protocol [51]. To determine the *K*_m ornithine_ and *k*_cat_ values, the reaction mixture contained various concentrations of ornithine, 0.2 mM PLP, 30 mm Tris-HCl (pH 7.8) and 0.4 ml of CO_2_-L3K assay buffer (12.5 mm phosphoenolpyruvate, 0.4 unit/ml microbial phosphoenolpyruvate carboxylase, 4.1 units/ml mammalian malate dehydrogenase and 0.6 mm NADH analog) at a final volume of 0.5 ml. The reaction commenced when the ODC enzyme was added to the assay mixture; simultaneously, the decrease in the absorbance at 405 nm was continuously recorded with a Perkin-Elmer Lambda-25 spectrophotometer. Using this coupled assay method under these conditions, 1 mol of CO_2_ was formed, and 1 mol of NADH analog was oxidized. An absorption coefficient of 2,410 cm^−1^ m ^−1^ was used for the NADH analog in the calculations. All calculations were performed with Sigma Plot 10.0 software (Jandel, San Rafael, CA).

### Size distribution analysis by analytical ultracentrifugation (AUC)

Sedimentation velocity experiments were conducted using a Beckman Optima XL-A analytical ultracentrifuge. Sample (380 µl) and buffer (400 µl) solutions were separately loaded into the double sector centerpiece and set up in a Beckman An-50 Ti rotor. The experiments were performed at 293 K with a rotor speed of 42,000 rpm. The protein samples were monitored using the UV absorbance at 280 nm in continuous mode, with a time interval of 420 s and a step size of 0.002 cm. Multiple scans at different time points were fitted to a continuous size distribution model using the program SEDFIT [52]–[55]. All size distributions were solved at a confidence level of p = 0.95, a best-fit average anhydrous frictional ratio (*f/f_0_*) and a resolution N of 200 sedimentation coefficients between 0.1 and 20.0 S.

To precisely determine the dissociation constants, sedimentation velocity experiments were performed at three different protein concentrations for both WT and mutant ODC: 0.3, 0.6 and 0.9 mg/ml in 50 mM Tris-HCl buffer (pH 7.5). The AUC scans were performed at 293 K. To calculate the dissociation constants (*K*_d_), all sedimentation data were globally fit to a monomer-dimer equilibrium model using the program SEDPHAT [54]. The partial specific volumes of the proteins, the solvent densities and the viscosity were calculated by the software program SEDNTERP [56].

### Measurement of protein stability by differential scanning calorimetry (DSC)

The DSC experiments were performed using a MicroCal VP-DSC (GE Healthcare). ODC WT and mutant proteins were used in these DSC scans at a concentration of 0.75 mg/ml in 30 mM acetate buffer (pH 7.0). The samples were scanned from approximately 283 K to 383 K at a heating rate of 1 K per min. In all experiments, the reference cell of the calorimeter was filled with a buffer equivalent to the sample buffer. The baseline buffer values were subtracted from the protein scans, and the molar heat capacity was used in the data analysis. Origin scientific plotting software (GE Healthcare) was utilized to analyze all DSC data. Before the DSC curve was fitted, the left and right linear line segments were assigned to create the progress baseline. After the data were normalized, curve fitting was performed with adequate models provided by Origin software. In this case, the unfolding curves were analyzed by a non-two-state transition model, which obeys the Levenberg/Marquardt (LM) non-linear least square method. Based on the calorimetric profiles, two or three peaks were assigned to facilitate the data fit. Up to 100 LM iterations and 100 simplex iterations were alternatively performed during the fitting cycles. Finally, best fit was observed when the chi square value could no longer be reduced. In this manner, the melting temperatures were acquired.

## Supporting Information

Figure S1 Continuous sedimentation coefficient distribution of the human ODC_WT and various single dimer-interface mutants. (A) ODC_WT; (B) ODC_D35G; (C) ODC_D134A; (D) ODC_R165E; (E) ODC_K169A; (F) ODC_R277S; (G) ODC_K294A; (H) ODC_V322D; (I) ODC_D332E; (J) ODC_Y389D. M, monomer; D, dimer.
(TIF)

Figure S2 DSC scanning plot of the human ODC_WT and single mutants. The protein concentration was 0.75 mg/ml in 30 mM sodium acetate buffer (pH 7.0). (A) ODC_WT; (B) ODC_R165E; (C) ODC_D134A; (D) ODC_K169A; (E) ODC_R277S; (F) ODC_Y331S; (G) ODC_D332E; (H) ODC_K294A; (I) ODC_V322D; (J) ODC_Y389D.
(TIF)
